# Thickness Effects on the Martensite Transformations and Mechanical Properties of Nanocrystalline NiTi Wires

**DOI:** 10.3390/nano12244442

**Published:** 2022-12-14

**Authors:** Gulsharat A. Baigonakova, Ekaterina S. Marchenko, Marina A. Kovaleva, Ekaterina A. Chudinova, Alex A. Volinsky, Yi Zhang

**Affiliations:** 1 Laboratory of Superelastic Biointerfaces, National Research Tomsk State University, 36 Lenin Ave., 634045 Tomsk, Russia; 2 Department of Mechanical Engineering, University of South Florida, 4202 E. Fowler Ave. ENG030, Tampa, FL 33620, USA; 3 School of Materials Science and Engineering, Henan University of Science and Technology, Luoyang 471023, China

**Keywords:** NiTi, wire, superelasticity effect, martensitic transformation, mechanical properties, nanostructure

## Abstract

This paper studied the features of the martensitic transformations and mechanical properties of 40, 60, and 90 µm thick NiTi wires with nanocrystalline B2 structures. It was established that the wires were composites and consisted of a TiNi matrix and a TiO_2_ + TiNi_3_ surface layer. Structural methods showed that the wire matrix was formed by grains of up to 20 nm in size. The method of measuring the electrical resistivity during cooling and heating revealed a two-stage nature of the martensitic transformation. Cyclic loading–unloading demonstrated that all the samples exhibited superelasticity effects and completely restored their shape when unloaded from a 4–8% relative strain at room temperature. An increase in mechanical characteristics with respect to the wire thickness was experimentally established. This was due to the change in the composition of the TiNi matrix during drawing.

## 1. Introduction

Nitinol (NiTi) is a well-known shape-memory alloy, which is widely used in industry and biomedicine [1,2,3]. Nitinol has gained popularity due to its unique properties, such as shape memory, superelasticity, good biocompatibility, and corrosion resistance [4,5,6,7,8]. These properties have made it possible to develop a large number of functional NiTi structures implantable in the human body. In many such applications, NiTi is used in the form of thin wires with 10–500 µm thickness and has good mechanical compatibility, shape memory, superelasticity, and good wear and corrosion resistance [9].

NiTi wires are important medical materials used in endovascular surgery, orthodontics, and soft tissue implants in the form of stents, catheters, orthodontic archwires, and mesh structures. However, the literature review showed that the study of the mechanical properties of NiTi wires has been carried out using samples with a diameter larger than 500 µm [10,11,12,13]. There are very few references devoted to the study of the mechanical properties of thin wires with a diameter of 40–100 µm. Therefore, the purpose of this research was to study the mechanical behavior of thin wires with diameters of 40, 60, and 90 µm to evaluate the dependence of their deformation characteristics on the wire thickness and to study their fracture surfaces.

## 2. Materials and Methods

Ni_50_Ti_50_ alloys were smelted in an induction furnace, and the obtained 240 mm long ingots with 20 mm diameters were subjected to repeated thermomechanical processing by drawing with intermediate annealing at 450 °C to obtain wires with 40, 60, and 90 μm thicknesses in 4 stages:

1. Strand rolling of an ingot with a 20 mm diameter to a bar with a 7 mm thickness (20 cycles);
2. Rotary forging of the bar from 7 mm to 3.5 mm (7 cycles);
3. Cold wire drawing from 3.5 mm to 0.5 mm (25 cycles);
4. Hot wire drawing with intermediate annealing at 450 °C was carried out for 50–70 cycles from a 500 µm diameter to 90, 60, and 40 µm thicknesses.

The deformation behavior of the nitinol microwires was studied using 50 mm long samples and 40, 60, and 90 µm thick samples by uniaxial tension-to-failure and cyclic loading–unloading tension testing. Stress–strain diagrams of uniaxial tension-to-failure and cyclic tension loading–unloading were carried out at room temperature with a tension rate of 0.1 mm/s using an Instron 3969 universal tensile testing machine. Precision pneumatic grippers, namely pneumatic cord, yarn, and fine wire grips, for cord and yarn grips G13KP up to 200 N were used to fasten the microwires. The universal tensile testing machine had a 3 μm displacement and a 0.004 N load resolution. 

Images of the fracture surfaces and the outer surfaces of the NiTi wires were obtained using a scanning electron microscope (Tescan MIRA 3 LMU) operated at a 20 kV accelerating voltage. The characteristic martensitic transformation (MT) temperature was determined from the electrical resistivity curves obtained using the potentiometric method. A chromel–alumel thermocouple was attached to the sample to measure its actual temperature. Liquid nitrogen was used as a coolant for tests below room temperature down to −180 °C. Heating above room temperature was performed using an electrical heater.

## 3. Results

### 3.1. Structural Studies

**Figure 1:**
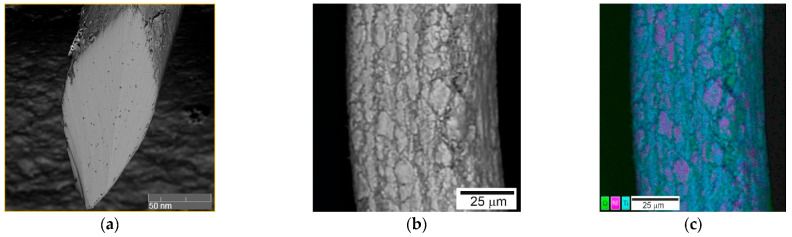
General view of the NiTi wire surface with a thickness of 60 µm: (**a**) cut wire; (**b**) wire surface; (**c**) distribution of elements on the wire surface.

The images of the surface morphology of the NiTi wires obtained using scanning electron microscopy showed that the wire consisted of a matrix and a rough surface layer, as shown in Figure 1. Nitinol wire with a diameter of less than 100 µm should be considered a composite material. A rough surface layer was formed on the wire surface during multiple cycles of intense plastic deformation by drawing with intermediate annealing in air at 450 °C. Interstitial impurities stimulated the matrix decomposition in the surface layers and the segregation of titanium to the surface [14,15,16].

**Figure 2:**
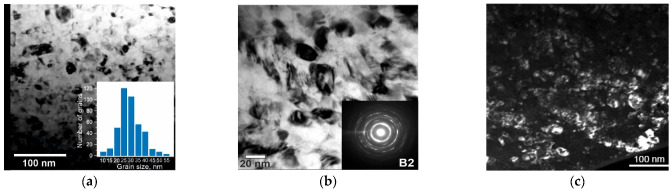
TEM images of the nanocrystalline structure of the NiTi wire with a thickness of 60 µm with the corresponding diffraction pattern from the B2 phase: (**a**) wire matrix structure and grain size distribution histogram; (**b**) magnified view of the wire matrix structure with the corresponding diffraction pattern; (**c**) dark-field image of the wire matrix structure.

The results of the transmission electron microscopy imaging of the wire matrix showed a nanocrystalline structure with a nanograin diameter of 15–65 nm and an average grain size of 25 nm, as shown in Figure 2a. The electron diffraction patterns showed that the NiTi wire matrix consisted of an austenitic B2 phase at room temperature, as shown in Figure 2b. Nanosized grains can lead to an increase in yield strength, which contributes to a greater accumulation of energy in the material during loading and enhances the deformation recovery by reversible MT when an external load is removed. Fragments of the subgrain structure in Figure 2c seen in the dark field did not exceed 20 nm. In some areas, clusters of particles were observed to be elongated in one direction due to the drawing process.

**Figure 3:**
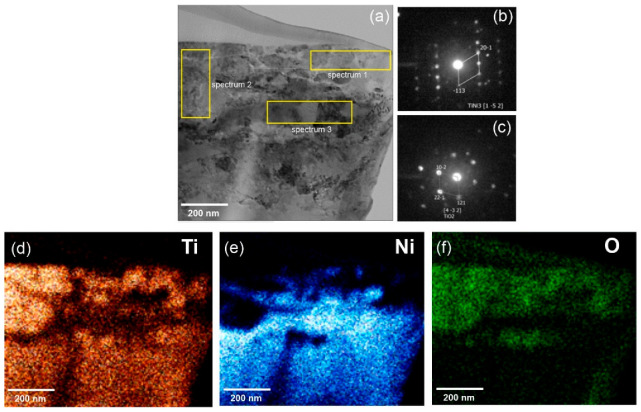
TEM image of a 60 μm wire section: (**a**) general view with selected areas for elemental analysis; (**b**) diffraction pattern of the selected area in spectrum 3; (**c**) diffraction pattern of the selected areas in spectra 1 and 2; (**d**–**f**) distribution maps of elements in the cross-section of the 60 μm thick NiTi wire.

A TEM study of the wire cross-section, which covered the areas of the matrix and the composite surface layer of the wire, showed a complex structure, as shown in Figure 3. The results of the elemental analysis showed that a non-continuous surface layer with a thickness of ~400 nm had regions that differed in contrast, indicating a different phase composition, as shown in Figure 3a. The bright areas shown were enriched in oxygen and titanium, while the dark areas were enriched in nickel. The diffraction patterns in Figure 3b,c and the elemental maps of these areas in Figure 3d–f identified the phases as TiO_2_ in spectra 1 and 2 and TiNi_3_ in spectrum 3. Such a mixed composition of the surface layer is characteristic of monolithic NiTi alloys subjected to annealing [15,16].

There are several research reports studying the complex surface oxidation processes in monolithic NiTi during annealing and the sequence of oxidation product formation [15,16,17]. A composite structure was formed, in which the outer layer was TiO_2_, and the inner layer was represented by phases enriched in nickel, Ni_3_Ti, and Ni(Ti). The TiO_2_ layer was formed first by the preferential oxidation of Ti from the NiTi matrix, resulting in a Ti-depleted layer under the outer TiO_2_ layer. The continued nickel enrichment of the titanium-depleted zone resulted in the formation of a TiNi_3_ layer during oxidation. A TiO_2_+Ni(Ti) composite layer was formed between the TiO_2_ and TiNi_3_ layers when the TiNi_3_ layer reached a critical thickness. The impact of intensive plastic deformation in the process of the wire production led to the mixing of the TiO_2_ and TiNi_3_ layers, as shown in Figure 3a, Table 1.

### 3.2. Mechanical Testing of NiTi Wires by Uniaxial Tension

**Figure 4:**
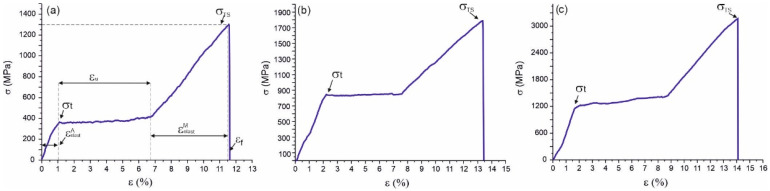
Engineering tensile stress–strain diagrams of NiTi wires with thicknesses of (**a**) 40 µm, (**b**) 60 µm, and (**c**) 90 µm tested to failure.

**Figure 5:**
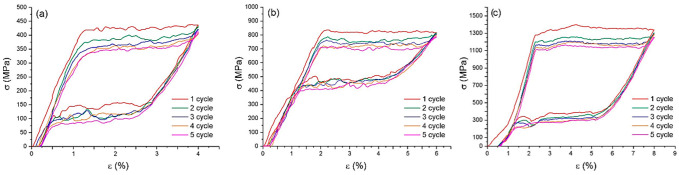
Engineering stress–strain diagrams of cyclic tension of NiTi wires with thicknesses of (**a**) 40 µm, (**b**) 60 µm, and (**c**) 90 µm.

Uniaxial tensile testing makes it possible to determine several important mechanical characteristics of a material, such as the critical stress of MT, the intervals of elastic and plastic deformation, and the tensile strength [5,6,18]. NiTi wires of different thicknesses (40, 60, and 90 µm) were tested by uniaxial tension-to-failure testing, as shown in Figure 4, and by using five loading–unloading cycles, as shown in Figure 5.

Three distinct regions were present in all the stress–strain diagrams. The initial linear region from a 0 to 2% strain was due to elastic deformation of the B2 austenite phase. The critical nominal stress of the MT, σ_t_, upon reaching which the austenite in the material became unstable and the nuclei of the martensite phase formed in the 40 µm, 60 µm, and 90 µm thick wire samples was about 350 MPa, 800 MPa, and 1200 MPa, respectively. The second region was the stress plateau at σ_t_ associated with the stress-induced transformation of austenite into martensite, which is often called the viscous flow region. The critical nominal stress plateau σ_t_ is associated with the onset of martensitic transformation, which extends by ε_m_ strain. In the process of martensitic transformation, the ε_m_ strain was 5.5% for the wires with a thickness of 40 µm and 60 µm and 7% for the wire with a thickness of 90 µm. The third region of these curves was due to linear hardening, which is associated with the elastic deformation of stress-induced martensite and was located in the strain range of 7–11.5% for the 40 µm thick wire, 7.5–13.5% for the 60 µm thick wire, and 8.5–14% for the 90 µm thick wire. For the samples under study, the tensile strength, σ_TS,_ was 1300 MPa for the 40 µm, 1800 MPa for the 60 µm, and 3150 MPa for the 90 µm thick wires. Based on the results of the tensile tests to failure, as shown in Table 2, the main mechanical characteristics of the wires were compiled, where σ_t_ is the nominal MT stress, σ_TS_ is the tensile strength, ε_f_ is the maximum strain at failure, ε_elast_ is the martensite elastic strain, and ε_m_ is the martensitic plateau strain width.

Cyclic stretching below the region of martensite linear hardening showed that all the samples exhibited superelasticity. This was expressed by an almost complete return of the 4–8% relative strain under the action of external cyclic loading at room temperature, as shown in Figure 5. A characteristic feature of the cyclic deformation of the tested samples was the presence of stress and strain hysteresis associated with internal friction losses and heating/cooling during reversible phase transformation. Under the cyclic stretching of the specimens with thicknesses of 40 µm, 60 µm, and 90 µm by 3%, 4%, and 6%, respectively, the irreversible deformation was 0.1–0.25% for the 40 and 60 µm thick wires and 0.5% for the 90 µm thick wires.

During cycling, a shift in strain was found with each subsequent cycle, and the stress–strain hysteresis values decreased. The value of the MT stress, σ_t,_ decreased by 14% at a thickness of 90 µm and by 18% at thicknesses of 40 µm and 60 µm. The decrease in the MT stress can be explained by the irreversible crystallographic reorientation of the martensite types. The applied stress caused the nucleation of martensite of different crystallographic orientations and the growth of certain types of martensite along the externally applied load direction. At the end of the transformation, the stress-induced martensite consisted of a single type, which is the ideal case. However, after each loading–unloading cycle, a portion of reoriented martensite remained, which is a source of internal stresses. In this regard, residual internal stresses appeared and increased with each new cycle, causing the earlier MT.

The nominal stress of MT decreased with decreasing the wire thickness. This was due to the influence of the composite surface layer, which did not undergo MT and led to a change in the concentration composition of the matrix and a decrease in its volume, which was responsible for the MT. The influence of surface layers on stress is complex and was difficult to determine due to several factors. On the one hand, the implementation phases in the form of TiO_2_ oxides, being brittle, crack at a 5–6% strain, which significantly exceeds their maximum deformation before failure [15]. On the other hand, the TiNi_3_ phase is an intermetallic compound that contributes to a higher stress than the critical stress for inducing martensite transformation in NiTi [16]. In addition, the modulus of elasticity of TiO_2_ and the intermetallic phases, Ti_2_Ni and TiNi_3,_ is much higher than the modulus of elasticity of the main TiNi phase, and the additive modulus of elasticity of the composite sample increases with their relative volume fraction [17]. In addition to all this, there was also a titanium-depleted zone, part of which had too low of a titanium content for martensitic transformation to occur.

### 3.3. Martensitic Transformations

**Figure 6:**
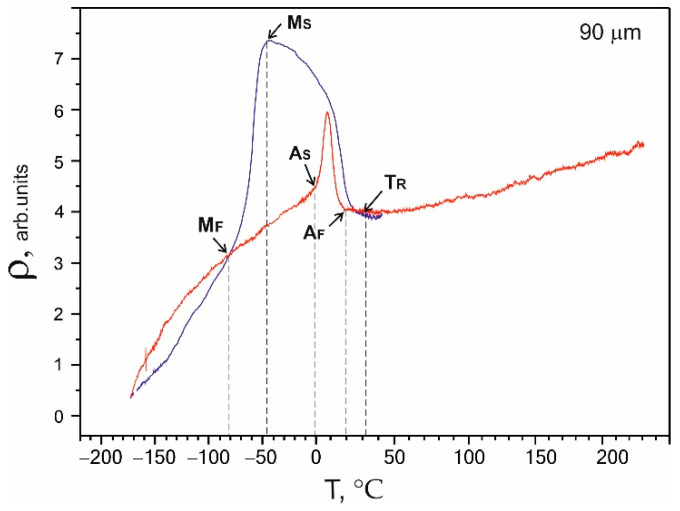
Temperature dependence of electrical resistivity ρ(T) for a 90 µm thick sample.

The main phenomena responsible for the manifestation of the superelasticity effects were thermoelastic martensitic transformations. The characteristic temperatures and the type of MT were determined based on the temperature dependence of the electrical resistivity ρ(T). The obtained electrical resistivity was typical for reversible MT В2↔R↔В19′ in binary NiTi alloys [19,20]. The arrows in Figure 6 mark the transformation temperatures. The deviation of the electrical resistivity from linearity during cooling reflected the beginning of the transition of the high-temperature В2 phase to the R phase and corresponded to the beginning temperature of the B2→R MT. A sharp decrease in the electrical resistivity during cooling corresponded to the M_S_ temperature of the direct R→B19′ MT onset. The M_F_ temperature on the ρ(T) curves corresponded to the end of the direct MT. A_S_ is the characteristic temperature of the reverse MT onset, while A_F_ is the characteristic temperature of the end of the reverse MT, which was determined similarly. M_F_ and A_F_ are defined at the points where the curves meet. 

A two-stage martensitic transition В2→R→B19′ was observed in the graph of the ρ(T) dependence for the sample with a 90 µm thickness, as shown in Figure 6, with the following characteristic temperatures: T_R_ = 31 °C, M_S_ = −49 °C, M_F_ = −80 °C, A_S_ = −2 °C, and A_F_ = 20 °C.

**Figure 7:**
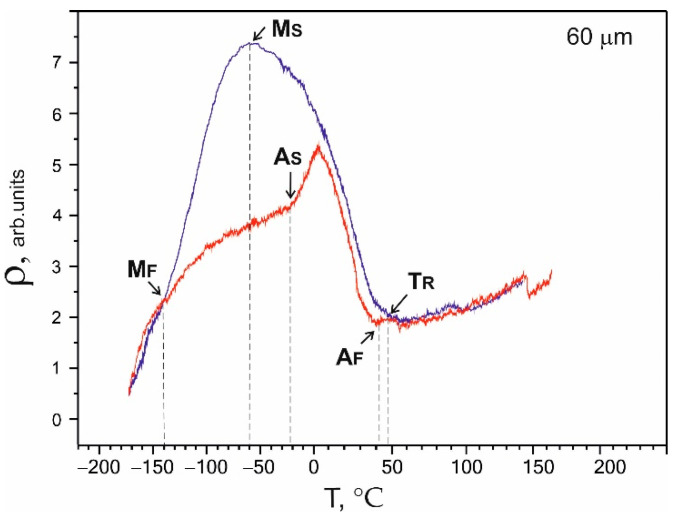
Temperature dependence of electrical resistivity ρ(T) for a 60 µm thick sample.

The characteristic temperatures for the 60 µm thick sample shown in Figure 7 were: T_R_ = 49 °C, M_S_ = −60 °C, M_F_ = −140 °C, A_S_ = −21 °C, and A_F_ = 40 °C.

**Figure 8:**
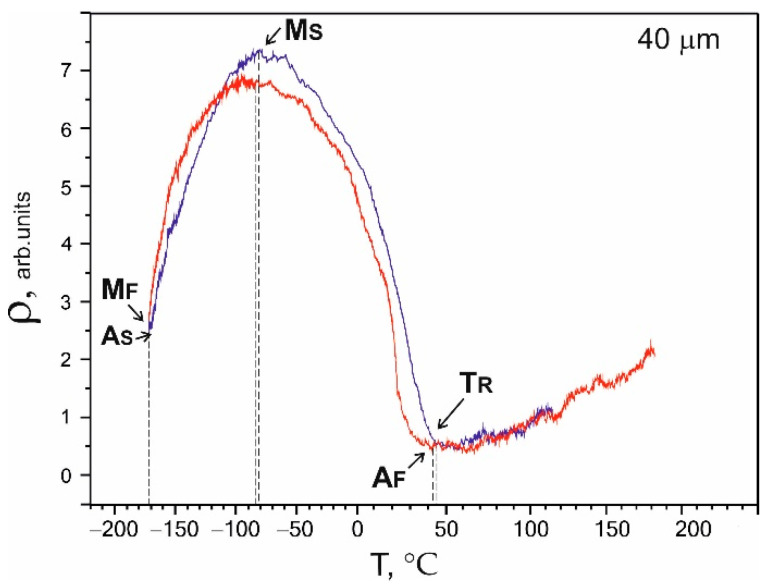
Temperature dependence of electrical resistivity ρ(T) for a 40 µm thick sample.

The same sequences of martensitic transformations were observed, as shown in Figure 6 and Figure 7, which were characteristic of nanocrystalline materials [19,21]. The dependence of the electrical resistivity on temperature ρ(Т) for the 40 µm thick sample in Figure 8 differed from the curves for the 60 μm and 90 µm wires shown in Figure 6 and Figure 7, respectively. A two-stage martensitic transition B2→R→B19′ was observed, and the characteristic temperatures of direct MT were graphically determined as T_R_ = 45 °C, M_S_ = −80 °C, and A_F_ = 42 °C. However, it was difficult to determine the temperature of the end of the direct martensitic transformation M_F_, since the temperature of the liquid nitrogen turned out to be insufficient to complete the direct martensitic transformation in the sample. The concentration of nickel in the thinnest sample increased significantly as a result of interaction with interstitial impurities and the decomposition of the TiNi phase.

The characteristic temperatures graphically determined from the temperature dependences of the electrical resistivity are summarized in Table 3.

The experimental results confirmed that the concentration composition of the TiNi matrix phase correlated with the thickness of the samples and the volume fraction of their surface layer. It was shown that the martensitic transition expanded and shifted to lower temperatures with decreasing the wire thickness.

**Figure 9:**
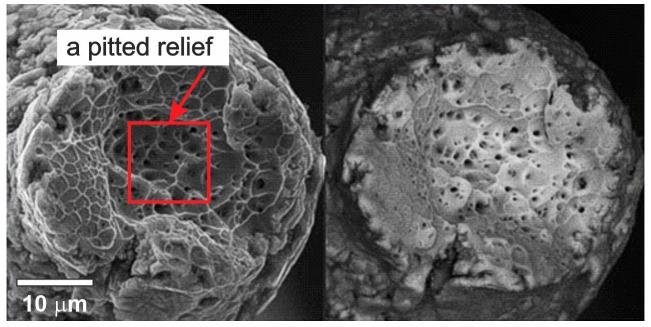
The fracture surfaces of a 40 µm thick NiTi wire.

SEM was used to study the fracture surfaces of the wires after a uniaxial tension failure. The obtained images of the 40 µm thick wire in Figure 9 clearly show the wire thinning at the point of rupture. The central part of the fracture surface was of a viscous type, as evidenced by the typical pitted relief with particles at the bottom of the cups as well as the combination of the deep center and the protruding periphery of the fracture zone.

**Figure 10:**
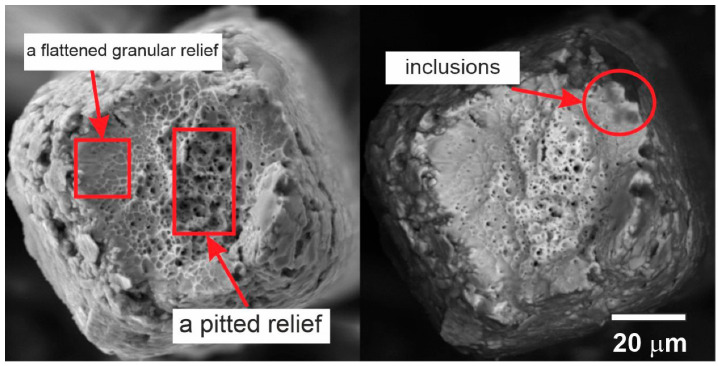
The fracture surfaces of a 60 µm thick NiTi wire.

Thinning was also found at the rupture site of the 60 µm thick sample shown in Figure 10. The revealed features of the relief allowed us to state that the shell enriched in non-metallic phases fractured due to the brittle separation mechanism, while the center, consisting of the viscous TiNi phase, fractured due to the ductile separation mechanism.

The thinning of the sample and the formation of a neck occurred due to the plastic shear of the viscous TiNi matrix. The fracture area of the shell was presented in the form of a roller with a size of 2–5 µm. The fracture surface also consisted of two zones: a flattened granular relief and a pitted relief, which had a typical appearance with particles at the bottom of the cups [22,23]. The fracture zone was located in a recess at the center of the destruction zone surrounded by zones of flattened relief. In this case, both parts had a viscous fracture type. The flattened relief was formed by plastic shear with the slow growth of a fracture crack, and the cup relief was formed as a result of fast viscous separation in the fracture zone [23]. 

**Figure 11:**
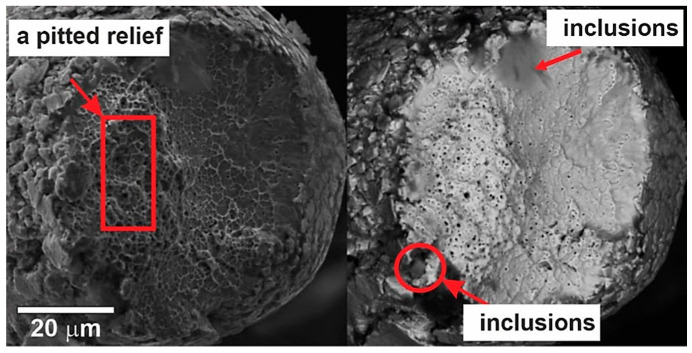
The fracture surfaces of a 90 µm thick NiTi wire.

Three large inclusions 5–10 µm in size were found at the opposite sides of the fracture zone, which served as sources for the formation of a crack and determined the ratio of the sizes of the crack formation zone and the fracture zone. The wire surface had a scaly relief, which was formed as a result of the plastic deformation of the grains of the viscous matrix during wire drawing. The size of the shell flakes was approximately 5–10 µm, and the size of the grains in the center of the matrix phase fracture was 2–5 µm. In the obtained images of a 90 µm thick wire shown in Figure 11, thinning of the wire was also present in the form of traces of plastic deformation at the rupture site.

The fracture zone had dimensions of 25–55 µm and was in a recess relative to the crack propagation zone. In this case, both parts had a viscous type of fracture. Two large non-metallic inclusions 15–25 µm and 10–15 µm in size were found at the opposite boundaries of the fracture zone, which served as sources for the formation of a crack and determined the dimensions of the fracture zone.

## 4. Conclusions

1. The microscopic studies showed that on the surface of the samples with a thickness of 60 μm, the interaction of interstitial impurities with the decomposition products of the TiNi matrix formed a surface layer of TiO_2_ oxides and TiNi_3_ intermetallic fragments of the decomposition of the TiNi matrix phase. During wire drawing, the impact of intensive plastic deformation in the presence of hydrocarbon lubricants and intermediate annealing in the air led to the formation of fragmented TiO_2_ surface layers mixed with fragmented TiNi_3_ intermetallic layers.
2. It was established that the main phase of the nitinol samples at room temperature before uniaxial tension was TiNi(B2) austenite.
3. The obtained temperature dependencies of electrical resistivity ρ(Т) for the NiTi samples with thicknesses of 40, 60, and 90 μm were characteristic of a two-stage martensitic transformation В2↔R↔B19′. It was established that the characteristic temperatures, M_S_, M_F_, and A_S,_ shifted to lower temperatures, and A_F_ shifted to a higher temperature as the sample thickness decreased to 40 μm.
4. The tests of the NiTi microwires with thicknesses of 40, 60, and 90 µm by uniaxial tension-to-failure and cyclic tension testing within the limits of reversible martensitic deformation showed that the NiTi samples with a thickness of 40–90 µm were characterized by superelastic behavior. An increase in the sample thickness led to an increase in the critical stresses of martensitic transformation from 300 to 1200 MPa, an increase in the ultimate tensile strength from 1300 to 3150 MPa, and an increase in the stress hysteresis width from 250 to 400 MPa. Such a correlation was due to a change in the composition of the TiNi matrix during wire drawing and annealing in air, the formation of a surface TiO_2_ oxide layer, and an intermetallic TiNi_3_ sublayer.

## Figures and Tables

**Table 1:** Results of elemental analysis in the surface layer of the 60 µm thick NiTi wire.

Element	Atomic %		
	Spectrum 1	Spectrum 2	Spectrum 3
O	65.7	67.39	5.71
Ti	33.33	31.98	20.81
Ni	1.97	0.63	73.48
Total	100	100	100

**Table 2:** Mechanical characteristics of NiTi wires.

Wire Thickness µm	σ_t_ MPa	σ_TS_ MPa	ε_f_ %	ε_elast_ %	ε_м_ %
40 ± 5	350 ± 35	1300 ± 130	11.5 ± 1	4.5 ± 0.4	5.5 ± 0.6
60 ± 5	800 ± 35	1800 ± 130	13.5 ± 1	5.9 ± 0.4	5.5 ± 0.6
90 ± 5	1200 ± 35	3150 ± 130	14 ± 1	5.4 ± 0.4	7.1 ± 0.6

**Table 3:** The characteristic temperatures of martensitic transformations in NiTi samples.

Wire Thickness µm	T_R_ °C	M_S_ °C	M_F_ °C	A_S_ °C	A_F_ °C
40 ± 5	45 ± 3	−80 ± 3	–	–	42 ± 3
60 ± 5	49 ± 3	−60 ± 3	−140 ± 3	−21 ± 3	40 ± 3
90 ± 5	31 ± 3	−49 ± 3	−80 ± 3	−2 ± 3	20 ± 3

## Data Availability

Not applicable.
